# The effect of continuous theta burst stimulation on antipsychotic-induced weight gain in first-episode drug-naive individuals with schizophrenia: a double-blind, randomized, sham-controlled feasibility trial

**DOI:** 10.1038/s41398-024-02770-w

**Published:** 2024-01-25

**Authors:** Dongyu Kang, Chuhan Song, Xingjie Peng, Guo Yu, Ye Yang, Chuwei Chen, Yujun Long, Ping Shao, Renrong Wu

**Affiliations:** Department of Psychiatry, National Clinical Research Center for Mental Disorders, and National Center for Mental Disorders, The Second Xiangya Hospital of Central South University, 410011 Changsha, Hunan China

**Keywords:** Schizophrenia, Physiology

## Abstract

Antipsychotic intake may induce weight gain in drug-naive individuals with schizophrenia, leading to poor compliance in clinical management. However, there is still a lack of effective approaches to treat or prevent this side-effect. Therefore, we conducted this pilot study to investigate the effect of continuous theta burst stimulation (cTBS), a non-invasive magnetic stimulation technique, on preventing olanzapine-induced weight gain. Thirty-nine first-episode drug-naive individuals with schizophrenia were randomly assigned to receive either the active or sham cTBS intervention for 25 sessions (5 times per day for 5 consecutive days). The primary outcomes were changes in body weight and body mass index (BMI). Secondary outcomes included psychiatric symptoms, eating behavior scales, behavior tasks, and metabolic measures. For the result, the body weight and BMI increased significantly in the sham group but not in the active group, with a significant group effect. The active group exhibited a selective increase in the cognitive restraint domain in the Three-Factor Eating Questionnaire (TFEQ-CR) and a decrease in stop-signal reaction time compared to the sham group. The effect of cTBS on body weight was mediated by TFEQ-CR. Our findings demonstrated the feasibility that cTBS intervention could be a potential method for preventing olanzapine-induced weight gain in drug-naive first-episode schizophrenia patients through enhancing cognitive restraint to food. Trial registration: clinical trial registered with clinicaltrials.gov (NCT05086133).

## Introduction

Schizophrenia is a mental disorder characterized by a set of syndromes including psychotic symptoms, negative symptoms, and cognitive impairment. It has a global prevalence of approximately 1% and significantly impacts individuals’ well-being and life quality, making it among the top 10 global causes of disability [1]. While most schizophrenia patients require lifelong antipsychotic treatment, almost all antipsychotics, particularly olanzapine, and clozapine, induce severe weight gain and insulin resistance [2]. These complications could lead to a heightened risk of cardiovascular disease and reduced life expectancy [3]. Although Olanzapine is more efficacious in treating overall symptoms [2], it can unfortunately cause the most severe metabolic side-effect [4]. The average weight gain of 10 weeks of olanzapine treatment was 4.15 kg [5]. In another study, individuals receiving olanzapine treatment gained weight rapidly in the first 12 weeks of treatment (mean gain, 7.3 kg), with the pace of weight gain slowed gradually, and the weight stabilized after a year (mean gain, 10.2 kg) [6]. Current guidelines recommend switching antipsychotic medication and lifestyle interventions as the primary strategy for managing these complications [7]. Our previous studies demonstrated lifestyle intervention [8], Metformin [9], Betahistine [10], and dietary fiber [11] are all effective in alleviating metabolic dysfunction and weight gain in individuals with schizophrenia. However, these approaches have limited effectiveness in clinical practice, highlighting the need for novel treatment options.

The mechanism underlying antipsychotics-induced weight gain is complex and involves several downstream pathways related to appetite regulation and eating behaviors [12]. Individuals with schizophrenia often experience increased appetite, which is a significant contributor to weight gain and dyslipidemia [13]. Moreover, decreased cognitive restraint on food intake, a domain of eating behavior often assessed by the Three-Factor Eating Questionnaire (TFEQ), is associated with higher body mass index (BMI) in individuals with schizophrenia [14]. This decreased cognitive control, combined with increased appetite, may contribute to weight gain in patients with schizophrenia. Moreover, the inhibitory control, as measured by the Stop-Signal test (SST), was negatively related to obesity and predicted weight change outcomes [15–17]. Both the diagnosis of schizophrenia [18] and antipsychotics are shown to impair inhibitory control [19] and potentially increase food intake together with increasing appetite [13]. Previous studies have identified the premotor area as a reflective region that exhibits altered activity during appetitive stimulations in schizophrenia patients [20], while also being related to cognitive restraint ability [18]. Specifically, the over-activation of the premotor area has been observed in schizophrenia with impaired inhibitory control [21], suggesting that the premotor area could serve as a potential target for treating weight gain and symptoms in this population.

Recent advances in non-invasive neuromodulation, such as Transcranial magnetic stimulation (TMS), offer a promising avenue for enhancing inhibition control ability by targeted regulation of cortical excitability in the premotor area. Studies have shown that suppression of neural activity in the primary motor cortex (M1 area) could significantly improve inhibitory control [22] while activating this region reduces cortical inhibition ability [23]. Meanwhile, a pilot study highlighted the M1 area as a potential cortical target for food cravings in healthy participants [24]. Theta burst stimulation (TBS) protocols, the developed patterns of repetitive TMS, have an excitatory effect if the stimulation is intermittent (iTBS), or a suppressive effect if the stimulation is continuous (cTBS) [25]. Currently, no study has evaluated the effect of cTBS over the left M1 area on preventing antipsychotic-induced weight gain.

We hypothesized that cTBS stimulation over the left M1 area could increase the inhibition control ability and prevent olanzapine-induced weight gain and eating behavior alteration. Here, we conducted a feasibility trial of cTBS stimulation over the left M1 region in drug-naive, first-episode individuals with schizophrenia to investigate its potential effects on olanzapine-induced weight gain and eating behavior alteration.

## Subjects and methods

### Study design and randomization

**Fig. 1:**
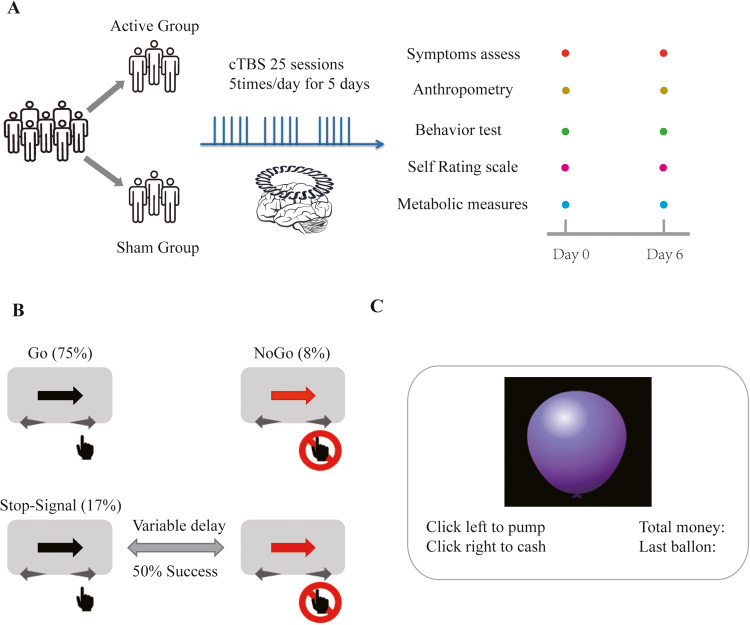
The concept of the research. **A** participants were randomized to an active group or sham group, receiving 25 sessions of cTBS intervention for 5 times per day for 5 continuous days. All evaluations were conducted at baseline and day 6. **B** There were Go trials (75%), NoGo trials (8%), and Stop-Signal trials (17%). In the Go trials, participants reacted to a left/right black arrow by pressing buttons with their right hand according to the arrow direction, using the index finger (for the left arrow) or middle finger (for the right arrow). In the NoGo trial, the participants were asked not to respond. In Stop-Signal trials, a response was initially cued by the left/right black arrow and changed to red after a Stop-Signal delay. The Stop-Signal delay was varied in each trial by using a step-up/down algorithm with a 250 ms initial estimate to maintain 50% successful inhibition. **C** In BART, a balloon is presented on the computer screen. Two options are provided to participants, the first option is to secure the amount of money in the current balloon (cash), and the second option is to take a risk and add more air to the balloon (pump).

A 5-day randomized, sham-controlled, double-blind clinical trial was conducted to evaluate the feasibility and effect of cTBS on antipsychotic-induced weight gain in first-episode drug naive individuals with schizophrenia. The trial was conducted at the Department of Psychiatry in Second Xiangya Hospital in Changsha, China. The study protocols were approved by the ethics committee of the Second Xiangya Hospital, Central South University, and the study was conducted in accordance with the Declaration of Helsinki. Written informed consent was obtained from all the participants after they received a complete description of the study. Participants were randomly assigned to receive either active or sham intervention while concurrently taking oral olanzapine monotherapy after the baseline assessment. Specifically, the first dose of olanzapine started at 7 p.m. after the baseline assessment, and the first cTBS simulation started in the morning of the following day. In total, 25 sessions of cTBS stimulation were delivered over 5 consecutive days, 5 sessions per day. A computer-based random number generator with a sealed randomization envelope was used for participant assignment. All participants were provided with identical meals with predetermined menus during the intervention. Outcome evaluation was conducted on baseline and day 6. All researchers were blind to the randomization except for the TMS coil operator. (Fig. 1A and supplement method).

### Participants

The sample size for the present study was estimated using previous studies of rTMS on eating disorders or obesity [26, 27], and one pilot study with tDCS [24]. All participants were recruited from the inpatient unit between November 2021 and Jun 2022 (Clinical Trial Registry: NCT05086133) by investigators from the Department of Psychiatry of the Second Xiangya Hospital at Central South University, China. Eligible participants were first-episode drug-naive individuals with schizophrenia, aged between 18 to 40 years old. All participants were diagnosed according to the criteria outlined in the DSM-V (Diagnostic and Statistical Manual of Mental Disorders, Fifth Edition) using the Chinese version of SCID by independent psychiatrists. Specifically, all participants had a course of the illness less than two years and had never received antipsychotics or physical treatment prior to the study. Exclusion criteria include (1) Diagnosed with other mental diseases in accordance with DSM-V; (2) Comorbid with other severe physiological diseases; (3) Pregnant or lactating women; (4) Contraindicated to TMS intervention.

### Metabolic and clinical outcomes

The primary outcomes were changes in body weight and BMI from baseline to 6 days. BMI was calculated as weight (in kg) divided by the height (in meters) squared. Secondary outcomes were the Positive and Negative Symptom Scale (PANSS), behavioral task parameters, Three-factor Eating Questionnaire (TFEQ), fasting serum glucose, triglyceride, total cholesterol, LDL, and HDL level. Specifically, TFEQ consists of three domains, cognitive restraint, uncontrolled eating, and emotional eating [28]. The PANSS scale, which was evaluated by an experienced, randomization-blinded psychiatrist in the ward, consists of three domains, positive symptoms, negative symptoms, and general symptoms [29].

### Behavioral task design

The study employed a task that comprised randomly interleaved NoGo and Stop-Signal trials to examine both types of inhibition (Fig. 1B). In the Go trials, participants were required to respond to a left/right black arrow by pressing buttons with their right hand according to the arrow direction using the index finger (left arrow) or middle finger (right arrow). In the NoGo trials, participants were required to withhold their response to the red left/right arrow. In Stop-Signal trials, a response was initially cued by the left/right black arrow but the arrow color changed to red after a Stop-Signal delay. The participants were instructed to refrain from responding to the stop signal [30]. The study measured six behavioral parameters of interest: namely Mean Go reaction time (GoRT), Stop-Signal mean reaction time (SSRT), Stop-Signal delay (SSD), Target accuracy, Go trial accuracy, and NoGo Accuracy [31].

The study also employed the Balloon Analogue Risk Task (BART), where a balloon representing a small monetary value was displayed on the computer screen in each trial (Fig. 1C). The participants were presented with two options. The first option was to secure the amount of money in the current balloon to the virtual bank account by pressing the right click on the mouse (cash), and the second option was to add more money and air to the balloon by pressing the left click on the mouse (pump) but taking the risk of bursting the balloon and losing the money. There were a total of 30 balloons in the task [32], and the study measured the Re-parameterized version of the BART model with 4 parameters: phi, the prior belief of balloon not bursting; eta, updating rate; gam, risk-taking parameter; tau, inverse temperature. The detailed task design has been published elsewhere [26], and so have the modeling descriptions [33].

### Transcranial magnetic stimulation intervention

The study employed a cTBS intervention, consisting of 25 sessions delivered over 5 consecutive days to the left primary motor cortex (M1 area), defined as the point of maximal abductor pollicis brevis stimulation. In each cTBS session, a total of 600 pulses over 200 bursts were delivered using a protocol of 50 Hz bursts of three pulses every 200 ms lasting 40 s, with an intensity of 80% of the individual resting motor threshold (RMT). The RMT intensity was measured as the minimum stimulus required to induce contraction of the right thumb at least 5 out of 10 times. The sham stimulation was performed in the same settings but with the coil tilted 90 degrees to the scalp to avoid real stimulation of the motor cortex while causing a similar skin sensation and sound.

### Statistical analysis

The study conducted an intent-to-treat analysis as randomized. Continuous variables were presented as mean and SD, and categorical variables were presented as ratios. One-way ANOVA was used for baseline comparison and within-group comparison of continuous variables, and the Chi-squared test was conducted for categorical variables. Repeated-measures analysis and Linear Mixed-Effects Models were used to compare group differences over time. The change in outcomes after the intervention was compared using the one-way ANOVA test. The Pearson correlation test was conducted to examine potential monotonic associations between variables. A simple mediation model was used for mediation analysis. The results were considered statistically significant if a two-tailed P value was <0.05. All analyses were conducted using the Statistical Package for Social Sciences, version 23 (SPSS Inc, Chicago, Illinois), PROCESS macro package in SPSS, and customized R script.

## Results

### Participants and baseline measures

Out of the 50 participants assessed for eligibility, 39 were randomized to either the sham or active intervention group. Of those, 19 participants in the active group and 20 participants in the sham group received the intervention. Two participants in the active group didn’t complete the intervention due to changing antipsychotics, while one participant in the sham group withdrew consent. As a result, data analysis included 17 participants in the active group and 19 participants in the sham group (92.3%) (Supplement Fig. 1). At baseline, the two groups had no significant difference in demographic, anthropometric, and metabolic measures, PANSS score, or the Three-Factor Eating Questionnaire (Supplement Table 1). Similarly, there were no significant differences in baseline behavior parameters between the two groups (Supplement Table 2).

### Weight and BMI

**Fig. 2:**
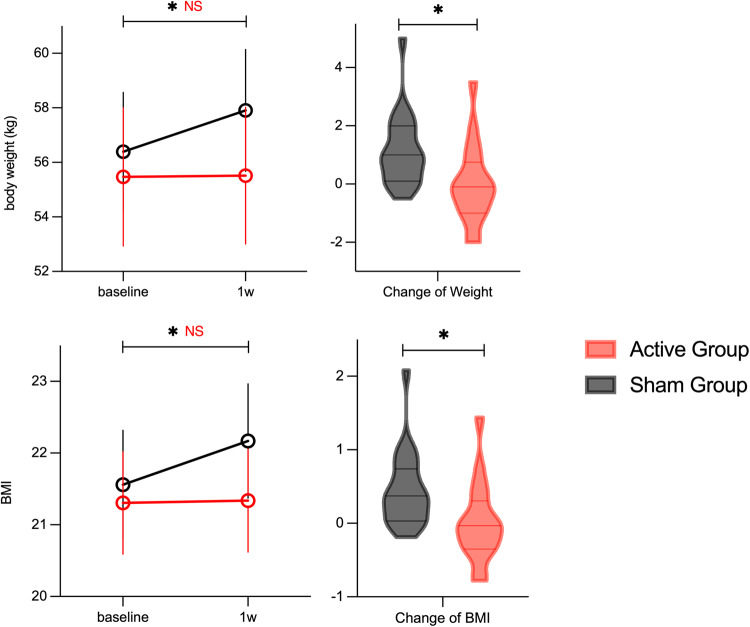
The effect of rTMS on weight and BMI. BMI, body mass index; In the sham group, the body weight and BMI increased significantly. There was a significant difference in body weight and BMI change between the two groups. ^*^p < 0.05.

In the active group, the body weight (mean of change = −0.01 kg, SD = 1.37) and BMI (mean of change = 0.01, SD = 0.54) did not alter significantly after 5 days of intervention. In contrast, in the sham group, the body weight (mean of change = 1.13 kg, SD = 1.23, *p* < 0.001, *η*^2^ = 0.470) and BMI (mean of change = 0.45, SD = 0.51, *p* < 0.001, *η*^2^ = 0.450) increased significantly. As illustrated in Fig. 2, the sham group had a greater increase in body weight and BMI compared to the active group. There was a significant difference in change in body weight (*F* = 7.503, *p* = 0.009, *η*^2^ = 0.17) and BMI (*F* = 6.850, *p* = 0.013, *η*^2^ = 0.16) between the two groups (Supplement Table 3). In the linear mixed effect model analysis, which takes each individual as a random effect in the formula, there was a significant time-by-group effect (*t*-value = −2.739, *p* = 0.009) in body weight, and BMI (*t*-value = −2.617, *p* = 0.0013) as well.

### Clinical symptoms

The clinical symptoms assessed by PANSS improved significantly in both groups. In the active group, the total score of PANSS (mean of change = −19.76, SD = 10.89, *p* < 0.001, *η*^2^ = 0.78), positive symptoms score (mean of change = −7.71, SD = 4.43, *p* < 0.001, *η*^2^ = 0.76) and general symptoms score (mean of change = −9.29, SD = 6.86, *p* < 0.001, *η*^2^ = 0.66) decreased significantly, although the negative symptoms score did not demonstrate a significant decrease (*p* = 0.053). Similarly in the sham group, the total score of PANSS (mean of change = −8.95, SD = 11.44, p = 0.002, *η*^2^ = 0.39), positive symptoms score (mean of change = −2.65, SD = 5.44, *p* = 0.029, *η*^2^ = 0.25) and general symptoms score (mean of change = −5.10, SD = 5.29, *p* < 0.001, *η*^2^ = 0.49) decreased significantly, but not the negative symptoms score (*p* = 0.276). The active group showed a significantly better improvement in total score (*F* = 8.584, *p* = 0.006, *η*^2^ = 0.20), positive symptoms score (*F* = 9.384, *p* = 0.004, *η*^2^ = 0.21), and general symptoms score (*F* = 4.402, *p* = 0.043, *η*^2^ = 0.11) than the sham group (Supplement Table 3).

### Behavior test and TFEQ

**Fig. 3:**
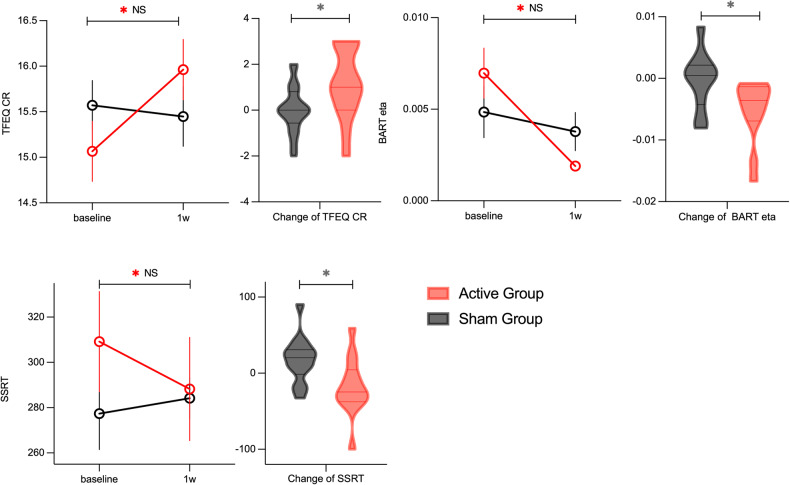
The effect of cTBS on TFEQ and behavior test. TFEQ-CR cognitive restraint domain in Three-factor Eating Questionnaire, BART eta updating rate in Balloon Analogue Risk Task, SSRT stop-signal reaction time; The cognitive restraint domain increased significantly while the BART updating rate and SSRT decreased significantly after intervention in the active group but not in the sham group; There’s a significant between-group difference in the cognitive restraint domain, BART updating rate, and SSRT. ^*^p < 0.05.

As shown in Fig. 3, the combined Stop-Signal and the Go-NoGo task result showed a decrease in the mean SSRT in the active group (mean of change = −17.74, SD = 37.92, *p* = 0.072) and an increase in the sham group (mean of change = 17.70, SD = 30.60, *p* = 0.074) post-intervention, with a significant difference between two groups (*F* = 7.192, p = 0.013, *η*^2^ = 0.19). These findings suggest a potential enhancing effect of cTBS and a deteriorating effect of antipsychotics on inhibition control. Other parameters in the task, such as GoRT, SSD, Target Accuracy, Go Accuracy, and NoGo Accuracy, were not altered after the intervention, and no significant differences were found between groups. In the BART test, there was a significant decrease in prior belief in the sham group (mean of change = −0.008, SD = 0.01, *F* = 11.297, *p* = 0.008, *η*^2^ = 0.56), and a significant decrease in updating rate in the active group (mean of change = −0.01, SD = 0.003, *F* = 20.444, *p* = 0.001, *η*^2^ = 0.65) after the intervention. The difference in the change in updating rate between the two groups was significant (Supplement Table 4).

The cognitive restraint domain in TFEQ increased significantly after the intervention in the active group (mean of change = 1.00, SD = 1.52, *F* = 6.324, *p* = 0.026, *η*^2^ = 0.33), but not in the sham group. There was a significant difference in the change in the cognitive restraint domain between the two groups (*F* = 4.859, *p* = 0.035, *η*^2^ = 0.13) (Supplement Table 3).

### Glucose and lipid metabolism

There were no significant changes in fasting glucose, triglyceride, total cholesterol, HDL, or LDL levels within both groups before and after intervention. There was no significant difference between the two groups (Supplement Table 3).

### Correlation between outcomes and the mediation effect model

**Fig. 4:**
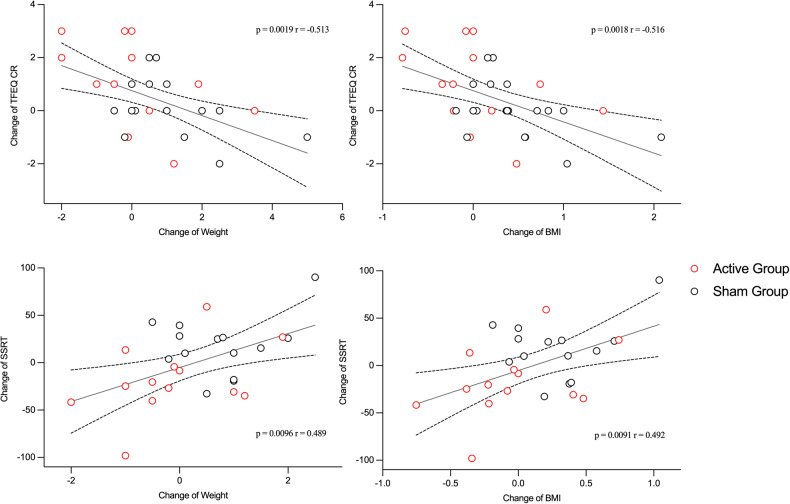
The correlation test between significant measures. TFEQ-CR, cognitive restraint domain in Three-factor Eating Questionnaire. SSRT stop-signal reaction time.

As demonstrated in Fig. 4, after the intervention, the change of cognitive restraint domain in TFEQ was negatively correlated to the change in BMI (*r* = −0.516, *p* = 0.0018) and body weight (*r* = −0.513, p = 0.0019), while the change of SSRT was positively correlated to the change of BMI (*r* = 0.492, p = 0.0091) and body weight (*r* = 0.489, *p* = 0.0096). Besides, the change in Go Accuracy was negatively correlated with the change in fasting glucose (*r* = −0.436, *p* = 0.030), and the improvement of negative symptoms score in PANSS was positively correlated to NoGo Accuracy (*r* = 0.537, *p* = 0.005) (Supplement Fig. 4c).

**Fig. 5:**
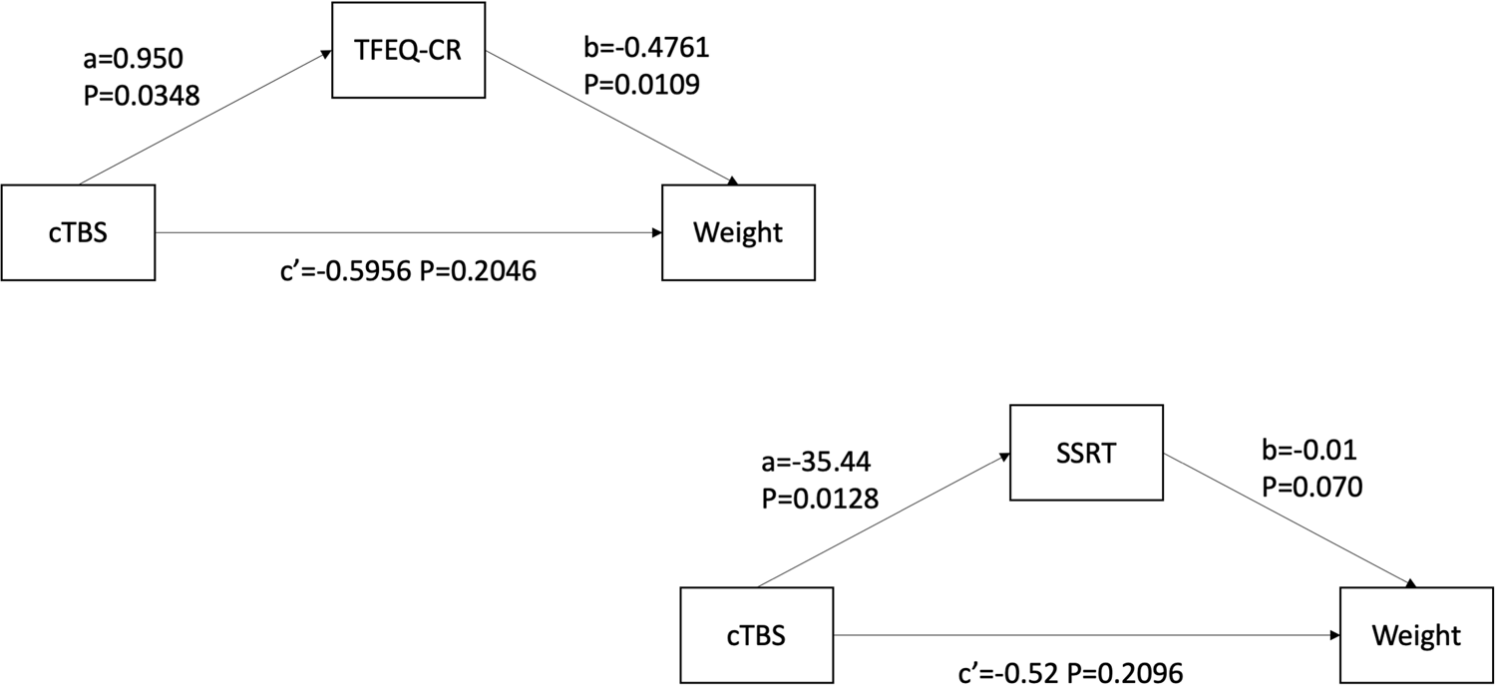
The mediation model of rTMS effect on weight. TFEQ-CR, cognitive restraint domain in Three-factor Eating Questionnaire. SSRT stop-signal reaction time, rTMS,= repetitive Transcranial magnetic stimulation. The “a” path reflects the direct effect of cTBS attenuation on the mediators. The “b” path reflects the direct effect of the mediator on the change in body weight. c’ indicates the direct effect of cTBS on the change in body weight. The number of a, b, and c’ refers to the coefficient of the corresponding variable in the model.

A simple mediation analysis was used to investigate the potential mediating mechanisms underlying the effect of cTBS intervention on body weight change [34]. The result suggested that TFEQ (TFEQ-CR) mediated the effect of cTBS on body weight. Controlling for TFEQ-CR, the direct effect of the intervention on body weight was insignificant (*p* = 0.2046, coefficient *c*’ = −0.4761). Furthermore, the analysis is also conducted using SSRT as the mediator, revealing a significant effect of cTBS on SSRT (*p* = 0.0128, coefficient *a* = −35.44), but no significant association between SSRT and body weight (*p* = 0.070, coefficient *b* = −0.01). Controlling for SSRT, the direct effect of rTMS on body weight was not significant (*p* = 0.2096, coefficient *c*’ = −0.52) (Fig. 5), indicating that inhibitory control is not a mediator in the relationship between rTMS and body weight.

## Discussion

The present study demonstrated that 25 sessions of cTBS targeting the left M1 area could prevent the weight gain induced by olanzapine in drug-naive first-episode individuals with schizophrenia. Meanwhile, the intervention increased cognitive restraint to food, as measured by the TFEQ-CR, and enhanced inhibitory control, as evidenced by decreased SSRT. The change in TFEQ-CR and SSRT were both related to the change in BMI and body weight. Furthermore, cognitive restraint mediated the effect of cTBS on body weight prevention.

Our results suggested that the effect of cTBS was mediated by the improved cognitive restraint on food, but not the direct effect of cTBS, on weight or BMI. Eating behavior has been a crucial factor in metabolism dysfunction in individuals with schizophrenia, and previous evidence suggested that a decreased cognitive restraint on food intake contributed to higher body mass index (BMI) [14]. By stimulating the key brain area involved in the neurocircuitry of inhibitory control, our study provided evidence for a potential ‘top-down’ regulation of the impaired inhibition of eating behavior in individuals with schizophrenia.

Our results on weight and BMI are consistent with one recent study by Su et al. In their study 47 participants with chronic schizophrenia received daily 10 Hz rTMS over dorsal lateral prefrontal cortex (DLPFC) for 4 weeks, resulting in a significant weight loss [35]. Another pilot study reported that cathodal transcranial direct current stimulation inhibited the M1 cortex activity and reduced self-reported hunger in healthy participants [24]. Furthermore, our results are also consistent with non-invasive neuromodulation studies targeting obesity. For instance, Kim et al. conducted a study on individuals with BMI ≥ 25, applying 10 Hz rTMS to the left DLPFC for 8 sessions within 2 weeks. After the treatment, they observed a greater weight loss in the active stimulation group (mean = −1.35 kg) [26]. In another study in 2019, an accelerated version of 32-session 10 Hz rTMS to left DLPFC within 4 weeks also resulted in a greater weight loss (mean = −2.75 kg) [36]. Even a lower dosage of rTMS, consisting of only 4 sessions of 10 Hz rTMS to left DLPFC could also affect body weight in obese individuals [37]. Ferrulli et al. reported that, compared with low-frequency 1 Hz deep rTMS and sham stimulation, only the 18 Hz deep rTMS decreased weight (mean = −1.3 kg) after the intervention [38].

rTMS has also been applied in studies targeting eating disorders. In 2018, Dalton et al. conducted a double-blind sham-controlled randomized clinical trial of rTMS in anorexia nervosa, showing that the active intervention resulted in a better mood, life quality, smaller BMI, and less severe eating disorder symptoms [27]. Ancillary analysis suggested rTMS improved self-controlled food choices after the intervention, which is similar to our results in TFEQ [39]. Moreover, our finding that rTMS intervention increased inhibitory control is also in line with the study by Guillaume, in which rTMS improved go/no-go task performance and cognitive impulsivity control in individuals with eating disorders [40]. However, most of the clinical trials targeted the prefrontal cortex exclusively.

Although, to our knowledge, no study has yet investigated the effect of cTBS on TFEQ or SSRT in individuals with schizophrenia, some evidence exists in healthy and obese populations. In healthy subjects, Obeso and colleagues have reported that cTBS over the pre-supplementary motor area (pre-SMA) significantly reduced SSRT compared to sham stimulation [41, 42]. Similarly, another study by Ji et al., also reported reduced SSRT over pre-SMA with iTBS intervention [43]. However, two studies failed to find any difference in SSRT after cTBS over pre-SMA [44, 45]. In the obese population, the correlation between eating behavior and inhibition control ability has been reported by numerous studies. For example, SSRT has been reported as a key indicator of weight or BMI status in obese adults [46]. Moreover, three studies have revealed that SSRT was correlated with or predicted weight loss during intervention in obesity [15–17]. Lastly, Previous research on TFEQ in schizophrenia indicated that all mean scores of three domains were higher in patients compared to healthy control. Specifically, the higher score in the cognitive restraint domain was related to lower BMI [14]. Our data was in accordance with most of the previous findings in healthy or obese populations.

Unexpectedly, our study demonstrated a significantly better improvement in PANSS total score, positive symptoms score, and general symptoms score following cTBS intervention. The use of rTMS to reduce psychotic symptoms is still in debate. Recent meta-analyses suggested a moderate but inconsistent effect of rTMS in general [47]. Further evidence on the effect of cTBS on psychotic symptoms is required before any conclusion.

The present study has certain limitations. First, our sample size of drug-naive first-episode is limited, and 86% (31/36) of the participants were female. The generalizability of our findings to larger populations of individuals with schizophrenia should be cautious. Second, although all participants were provided with identical meals during the intervention, we didn’t record the precise energy consumed by each participant. Lastly, the entire trial duration was relatively short (5 days), and we did not conduct a follow-up assessment after the intervention, hence could not determine the duration of this intervention’s effect on weight gain prevention. Meanwhile, the difference in weight gain between active and sham groups was not clinically significant at the end of the study.

In summary, our study demonstrated the feasibility and potential of cTBS intervention as an effective prevention for olanzapine-induced weight gain by enhanced cognitive restraint in eating behavior in drug-naive first-episode individuals with schizophrenia. Future studies with well-designed follow-ups, neuronavigation TMS settings, and larger sample sizes should be promising.

### Supplementary information

Supplement tables and figures

## Data Availability

The data will be available only to those whose proposition on the use of the data, for scientific research specified in a proposal, has been approved by both corresponding authors [RRW] and [PS].
