# Supplementary material for: The effect of continuous theta burst stimulation on antipsychotic-induced weight gain in first-episode drug-naive individuals with schizophrenia: a double-blind, randomized, sham-controlled feasibility trial
Source: Transl Psychiatry. 2024 Jan 25;14:61. doi: 10.1038/s41398-024-02770-w (PMC10810827; doi:10.1038/s41398-024-02770-w)
Supplement: Supplementary file 1 — Supplement tables and figures [file 41398_2024_2770_MOESM1_ESM.docx]

**The effect of continuous theta burst stimulation on antipsychotic-induced weight gain in first-episode drug-naïve individuals with schizophrenia: a double-blind, randomized, sham-controlled feasibility trial**

Dongyu Kang, M.D.^1^, Chuhan Song, M.D.^1^, Xinjie Peng, M.D. ^1^, Guo Yu, MSc.^1^, Ye Yang, M.D.^1^, Chuwei Chen, M.D^1^, Yujun Long, M.D.^1^, Ping Shao M.D. PhD^1, *^, Renrong Wu, M.D. PhD.^1, *^

**Affiliation/address:**

1. Department of Psychiatry, National Clinical Research Center for Mental Disorders, and National Center for Mental Disorders, The Second Xiangya Hospital of Central South University, Changsha 410011, Hunan, China.

***Corresponding author and E-mail address:**

Renrong Wu, MD PhD

National Clinical Research Center for Mental Disorders

Department of Psychiatry of the Second Xiangya Hospital

Central South University

Changsha, Hunan

China

E-mail: wurenrong@csu.edu.cn

Tel: +86 15874179855； Fax: +86 73185295214

Ping Shao MD PhD

National Clinical Research Center for Mental Disorders

Department of Psychiatry of the Second Xiangya Hospital

Central South University

Changsha, Hunan

China

E-mail: [Shp97jw@163.com](mailto:Shp97jw@163.com)

Tel: +86 13755112445； Fax: +86 73185295214

**Keywords**: continuous theta burst stimulation (cTBS); eating behavior; weight gain; schizophrenia; inhibition control; antipsychotics.

Words: 3605 Figure: 5

Supplement Table 1 baseline characteristics of demographic, metabolic, and rating scales.

|  | Active | Sham | F df=1 | P |
| --- | --- | --- | --- | --- |
| Age | 26.88(8.462) | 24.55(6.216) | 0.990 | 0.326 |
| Gender (female, male) | (14, 3) | (17,2) | Chi-square 0.380 | Fisher exact test 0.650 |
| Course (month) | 12.66(19.11) | 20.00(28.92) | 0.779 | 0.383 |
| Olanzapine Dose | 13.13(4.03) | 15.26(4.57) | 2.122 | 0.155 |
| Height (m) | 1.61(0.07) | 1.61(0.08) | 0.016 | 0.900 |
| Weight (kg) | 56.21(10.64) | 56.71(10.56) | 0.021 | 0.885 |
| BMI | 21.51(3.00) | 21.70(3.71) | 0.028 | 0.868 |
| Fasting Glucose | 4.35(0.55) | 4.32(0.43) | 0.027 | 0.871 |
| TY | 1.11(0.39) | 1.11(0.53) | 0.001 | 0.990 |
| TC | 4.08(0.70) | 4.08(0.70) | 0.001 | 0.972 |
| LDL | 2.40(0.43) | 2.32(0.55) | 0.195 | 0.662 |
| HDL | 1.31(0.33) | 1.36(0.34) | 0.177 | 0.677 |
| PANSS Total | 77.24(9.44) | 72.71(13.81) | 1.319 | 0.258 |
| PANSS Positive | 19.88(3.89) | 17.05(5.06) | 3.476 | 0.071 |
| PANSS Negative | 20.76(5.87) | 21.48(6.55) | 0.122 | 0.729 |
| PANSS general | 36.59(6.35) | 34.81(5.15) | 0.909 | 0.347 |
| TFEQ total | 56.11(7.25) | 56.13(6.01) | 0.001 | 0.992 |
| TFEQ UE | 24.00(4.23) | 23.57(4.11) | 0.100 | 0.754 |
| TFEQ CR | 15.09(1.28) | 15.64(1.23) | 1.690 | 0.202 |
| TFEQ EE | 17.24(2.25) | 16.92(2.48) | 0.168 | 0.684 |

Supplement Table 2. baseline characteristic of behavior tests.

|  | Active | Sham | F df=1 | P |
| --- | --- | --- | --- | --- |
| **SST** | | | | |
| GoRT | 521.57(153.90) | 474.81(62.70) | 1.246 | 0.274 |
| SSD | 212.45(99.23) | 197.34(89.62) | 0.192 | 0.665 |
| SSRT | 309.11(83.05) | 277.47(63.50) | 1.395 | 0.248 |
| Target Accuracy | 0.49(0.05) | 0.45(0.07) | 3.005 | 0.094 |
| Go Accuracy | 0.84(0.13) | 0.83(0.12) | 0.071 | 0.793 |
| NoGo Accuracy | 0.52(0.48) | 0.33(0.44) | 1.236 | 0.276 |
| **BART** | | | | |
| Prior belief | 0.98(0.01) | 0.99(0.01) | 3.393 | 0.078 |
| Updating rate | 0.007(0.003) | 0.01(0.01) | 0.023 | 0.880 |
| Risk taking | 0.54(0.30) | 0.50(0.31) | 0.154 | 0.698 |
| Inverse temperature | 0.22(0.16) | 0.24(0.23) | 0.081 | 0.778 |

Supplement Table 3 Effect of rTMS intervention on metabolic indexes and symptoms

|  | baseline | | 1-week | | ∆ of 1-week | | Active 1w RMT | | Sham 1w RMT | | Group difference | |
| --- | --- | --- | --- | --- | --- | --- | --- | --- | --- | --- | --- | --- |
|  | Active | Sham | Active | Sham | Active | Sham | F | P | F | P | F | P |
| Weight (kg) | 56.21(10.64) | 56.71(10.56) | 56.19(10.56) | 57.84(10.47) | -0.01(1.37) | 1.13(1.23) | 0.001 | 0.972 | 18.642 | <0.001 | 7.503 | 0.009 |
| BMI | 21.51(3.00) | 21.70(3.71) | 21.51(3.05) | 22.14(3.72) | 0.01(0.54) | 0.45(0.51) | 0.002 | 0.963 | 17.174 | <0.001 | 6.850 | 0.013 |
| Glu | 4.35(0.55) | 4.32(0.43) | 4.06(0.55) | 4.24(0.58) | -0.23(0.60) | -0.14(0.54) | 2.188 | 0.161 | 1.264 | 0.276 | 0.196 | 0.661 |
| TY | 1.11(0.39) | 1.11(0.53) | 1.09(0.63) | 1.15(0.61) | -0.01(0.71) | 0.07(0.61) | 0.001 | 0.970 | 0.296 | 0.593 | 0.126 | 0.725 |
| TC | 4.08(0.70) | 4.08(0.70) | 4.12(0.75) | 4.01(0.73) | 0.09(0.49) | -0.05(0.72) | 0.418 | 0.529 | 0.111 | 0.743 | 0.388 | 0.537 |
| LDL | 2.40(0.43) | 2.32(0.55) | 2.43(0.55) | 2.36(0.52) | 0.10(0.33) | 0.06(0.52) | 1.285 | 0.277 | 0.247 | 0.624 | 0.074 | 0.788 |
| HDL | 1.31(0.33) | 1.36(0.34) | 1.31(0.34) | 1.31(0.37) | -0.02(0.20) | -0.06(0.31) | 0.240 | 0.633 | 0.682 | 0.419 | 0.097 | 0.757 |
| TFEQ SUM | 56.11(7.25) | 56.13(6.01) | 56.37(3.60) | 54.32(6.42) | 0.26(7.02) | -1.81(5.43) | 0.023 | 0.882 | 2.330 | 0.143 | 1.047 | 0.313 |
| TFEQ UE | 24.00(4.23) | 23.57(4.11) | 23.78(2.22) | 22.43(3.26) | -0.22(4.46) | -1.14(3.43) | 0.042 | 0.841 | 2.327 | 0.143 | 0.519 | 0.476 |
| TFEQ CR | 15.09(1.28) | 15.64(1.23) | 15.96(1.33) | 15.48(1.49) | 1.00(1.52) | 0.05(0.10) | 6.324 | 0.026 | 0.182 | 0.674 | 4.859 | 0.035 |
| TFEQ EE | 17.24(2.25) | 16.92(2.48) | 16.55(1.62) | 15.99(2.58) | -0.68(2.29) | -0.92(1.92) | 1.512 | 0.237 | 4.822 | 0.040 | 0.122 | 0.729 |
| PANSS T | 77.24(9.44) | 72.71(13.81) | 57.47(9.43) | 63.67(12.84) | -19.76(10.89) | -8.95(11.44) | 56.010 | <0.001 | 12.249 | 0.002 | 8.584 | 0.006 |
| PANSS P | 19.88(3.89) | 17.05(5.06) | 12.18(2.58) | 13.48(4.65) | -7.71(4.43) | -2.65(5.44) | 51.515 | <0.001 | 5.698 | 0.029 | 9.384 | 0.004 |
| PANSS N | 20.76(5.87) | 21.48(6.55) | 18.00(4.47) | 20.90(5.51) | -2.76(5.45) | -1.20(4.77) | 4.376 | 0.053 | 1.257 | 0.276 | 0.865 | 0.359 |
| PANSS G | 36.59(6.35) | 34.81(5.15) | 27.29(4.70) | 29.29(6.25) | -9.29(6.86) | -5.10(5.29) | 31.181 | <0.001 | 18.586 | <0.001 | 4.402 | 0.043 |

Repeated measure test within group.

Supplement Table 4 Effect of rTMS intervention on SST and BART

|  | baseline | | 1-week | | ∆ of 1-week | | Active 1w RMT | | Sham 1w RMT | | Group difference | |
| --- | --- | --- | --- | --- | --- | --- | --- | --- | --- | --- | --- | --- |
|  | Active | Sham | Active | Sham | Active | Sham | F | P | F | P | F | P |
| SST | | | | | | | | | | | | |
| GoRT | 521.57(153.90) | 474.81(62.70) | 496.43(177.64) | 454.75(90.84) | -52.31(95.53) | -24.36(79.99) | 2.372 | 0.149 | 1.298 | 0.275 | 0.683 | 0.416 |
| SSD | 212.45(99.23) | 197.34(89.62) | 208.04(122.09) | 170.55(75.68) | -26.88(58.49) | -45.62(97.88) | 2.744 | 0.124 | 3.042 | 0.105 | 0.358 | 0.555 |
| SSRT | 309.11(83.05) | 277.47(63.50) | 288.39(85.35) | 284.20(36.49)) | -17.74(37.92) | 17.70(30.60) | 3.898 | 0.072 | 3.785 | 0.074 | 7.192 | 0.013 |
| Target Accuracy | 0.49(0.05) | 0.45(0.07) | 0.46(0.08) | 0.44(0.12) | -0.05(0.07) | -0.03(0.12) | 5.241 | 0.041 | 1.021 | 0.331 | 0.098 | 0.757 |
| Go Accuracy | 0.84(0.13) | 0.83(0.12) | 0.82(0.18) | 0.86(0.11) | -0.03(0.11) | -0.001(0.13) | 0.834 | 0.379 | 0.001 | 0.987 | 0.369 | 0.549 |
| NoGo Accuracy | 0.52(0.48) | 0.33(0.44) | 0.43(0.43) | 0.39(0.45) | -0.10(0.56) | 0.02(0.53) | 0.377 | 0.551 | 0.031 | 0.862 | 0.332 | 0.570 |
| BART | | | | | | | | | | | | |
| Prior belief | 0.98(0.01) | 0.99(0.01) | 0.98(0.01) | 0.98(0.01) | -0.002(0.01) | -0.008(0.01) | 0.890 | 0.366 | 11.297 | 0.008 | 2.879 | 0.105 |
| Updating rate | 0.007(0.003) | 0.01(0.01) | 0.001(0.001) | 0.003(0.003) | -0.01(0.003) | 0.0002(0.004) | 20.444 | 0.001 | 0.030 | 0.867 | 9.036 | 0.007 |
| Risk taking | 0.54(0.30) | 0.50(0.31) | 1.01(1.01) | 0.66(0.41) | 0.53(1.09) | 0.17(0.35) | 2.805 | 0.122 | 2.460 | 0.151 | 0.979 | 0.334 |
| Inverse temperature | 0.22(0.16) | 0.24(0.23) | 0.24(0.24) | 0.29(0.19) | -0.07(0.15) | 0.04(0.27) | 2.414 | 0.149 | 0.176 | 0.685 | 1.328 | 0.263 |

Repeated measure test within group.

Supplement Figure 1 study flowchart

**CONSORT 2010 Flow Diagram**

Finished intervention (n=17)

Changed medication (n= 2)

Allocated to Active intervention (n= 19)

♦ Received allocated intervention (n=19 )

Excluded (n=11)

♦  Not meeting inclusion criteria (n= 5)

♦  Declined to participate (n= 4)

♦  Other reasons (n= 2)

Randomized (n= 39)

Assessed for eligibility (n=50)

Analysed (n= 19)
♦ Excluded from analysis

(Didn’t finish 1 week intervention) (n=1)

Analysed (n= 17)
♦ Excluded from analysis

(Didn’t finish 1 week intervention) (n=2)

Finished intervention (n= 19)

Withdraw consent = (1)

Allocated to Sham intervention (n= 20)

♦ Received allocated intervention (n=20)

## Analysis

## Intervention

## Allocation

## Enrollment

Supplement Figure 2 Correlation Test Matrices at baseline in all participants.

Supplement Figure 3 Correlation of baseline measures to change of outcomes after treatment.

 Supplement Figure 3a Active Group Supplement Figure 3b sham group

 Supplement Figure 3c all participants

Supplement Figure 4 Correlation matrix of change of outcomes.

 Supplement Figure 4a Active Group Supplement Figure 4b Sham Group

Supplement Figure 4c All Participant

**Supplement of method**

(Day 0) The participants were recommended to participate in the study by psychiatry clinicians in the morning. For those participants who were interested, an interview would be scheduled for screening and consent. Written consent was obtained at the end of this interview and was required for collecting any further data. This process was generally finished before noon.

In the afternoon, the participants were interviewed for the Chinese version of the Structured Clinical Interview for DSM-5 Disorders (Michael Philips et al.), Positive and Negative Symptom Scale (PANSS), demographic information, and medical history by a randomization-blinded psychiatrist. This process usually takes two to three hours. Afterward, the participants were asked to finish the Three-factor Eating Questionnaire (TFEQ), a task that comprised randomly interleaved NoGo and Stop-Signal, and the Balloon Analogue Risk Task (BART). This process usually takes another one to two hours. Participants received their first dose of oral olanzapine medication at around 6 p.m. on the same day.

(Day 1) At 6 a.m. of the following day, fasting blood samples were collected by research nurses for the laboratory test of fasting serum glucose, triglyceride, total cholesterol, LDL, and HDL levels. The body weight and height were measured before breakfast. The first cTBS session was conducted at around 9 a.m. after breakfast, while the other 4 sessions were conducted around 10 a.m., 11 a.m. 2 p.m., and 3 p.m., with a one-hour interval between each session. The stimulation was repeated for another four consecutive days (Day 2 - Day 5), which in total consisted of 25 sessions of cTBS. The olanzapine was administrated orally at 6 p.m. each day.

(Day 6) On the following day of the last cTBS, which usually was 12 hours after the last session, the fasting blood sample was collected at 6 a.m. The body weight and height were measured before breakfast. The PANSS scale was then evaluated by a randomization-blinded psychiatrist in the ward. The TFEQ and two behavior tasks were conducted by a research assistant or PhD student during the rest of the day.

Supplement of correlation tests

At baseline, the correlation test indicated that the course of the disease was positively associated with negative symptoms score in PANSS (r= 0.568, p< 0.001) and negatively associated with positive symptoms score in PANSS (r= -0.349, p= 0.040). The olanzapine dosage was positively related to the uncontrollable eating domain (r= 0.497, p=0.003) and emotional eating domain (r= 0.401, p=0.019) in TFEQ (Supplement Figure 2).

To assess the potential for baseline measures to predict intervention outcomes, the Pearson correlation test was performed between all pairs of variables. Noteworthy results are: a significant positive correlation was found between the general symptom score in PANSS at the baseline and the increase in BMI (r= 0.619, p= 0.038), indicating a higher increase in BMI in patients with a more severe general symptom; And a negative correlation between the baseline SSRT and changes in total cholesterol, LDL, and HDL, suggesting a better inhibition control at the baseline is associated with a higher change in the metabolic outcomes. (Supplement Figure 3).
